# Role of Basic Fibroblast Growth Factor in Cancer: Biological Activity, Targeted Therapies, and Prognostic Value

**DOI:** 10.3390/cells12071002

**Published:** 2023-03-24

**Authors:** Alessio Ardizzone, Valentina Bova, Giovanna Casili, Alberto Repici, Marika Lanza, Raffaella Giuffrida, Cristina Colarossi, Marzia Mare, Salvatore Cuzzocrea, Emanuela Esposito, Irene Paterniti

**Affiliations:** 1 Department of Chemical, Biological, Pharmaceutical and Environmental Sciences, University of Messina, Viale Ferdinando Stagno d’Alcontres, 98166 Messina, Italy; 2 IOM Ricerca Srl, Via Penninazzo 11, 95029 Viagrande, Italy; 3 Istituto Oncologico del Mediterraneo, Via Penninazzo 7, 95029 Viagrande, Italy

**Keywords:** basic fibroblast growth factor (bFGF), fibroblast growth factor receptors (FGFRs), bFGF/FGFR axis, tumor microenvironment

## Abstract

Cancer is the leading cause of death worldwide; thus, it is necessary to find successful strategies. Several growth factors, such as vascular endothelial growth factor (VEGF), basic fibroblast growth factor (bFGF, FGF2), and transforming growth factor beta (TGF-β), are involved in the main processes that fuel tumor growth, i.e., cell proliferation, angiogenesis, and metastasis, by activating important signaling pathways, including PLC-γ/PI3/Ca^2+^ signaling, leading to PKC activation. Here, we focused on bFGF, which, when secreted by tumor cells, mediates several signal transductions and plays an influential role in tumor cells and in the development of chemoresistance. The biological mechanism of bFGF is shown by its interaction with its four receptor subtypes: fibroblast growth factor receptor (FGFR) 1, FGFR2, FGFR3, and FGFR4. The bFGF–FGFR interaction stimulates tumor cell proliferation and invasion, resulting in an upregulation of pro-inflammatory and anti-apoptotic tumor cell proteins. Considering the involvement of the bFGF/FGFR axis in oncogenesis, preclinical and clinical studies have been conducted to develop new therapeutic strategies, alone and/or in combination, aimed at intervening on the bFGF/FGFR axis. Therefore, this review aimed to comprehensively examine the biological mechanisms underlying bFGF in the tumor microenvironment, the different anticancer therapies currently available that target the FGFRs, and the prognostic value of bFGF.

## 1. Introduction

Fibroblast growth factors (FGFs) represent a family of heparin-binding growth factors which are involved in numerous biological processes, such as angiogenesis, wound healing, neurogenesis, cell differentiation, and migration, but also in different signal transduction processes [1]. In humans, numerous isoforms of FGFs (FGF1-FGF23) have been isolated; they are structurally related to each other and bind the four types of fibroblast growth factor receptors (FGFRs) identified in humans (FGFR1-FGFR4) [1].

FGFRs are expressed in many cell types, and they are recognized as tyrosine kinase receptors. Every FGF can bind different receptor subtypes, except for FGF1, which is the only one able to bind all the receptor subtypes (FGFR1-FGFR4) [2]. Moreover, the basic fibroblast growth factor (bFGF; FGF2) is the prototypical ligand among all the members, and it binds, like FGF1, all the receptor subtypes; in comparison, all the other FGFs show a heterogeneous selectivity [3]. Beyond the functions of the basic fibroblast growth factor (bFGF; FGF2) in biological processes, it also plays critical roles in the pathophysiological conditions promoting tumorigenesis [4].

In this context, bFGF secreted by tumor cells mediates several transduction signals and plays an influential role in the tumor environment [5].

These mechanisms underlie the development of resistance to chemotherapies and also suggest that bFGF has a reliable prognostic value [4].

There is a lot of compelling evidence regarding the alterations of bFGF signaling in the pathogenesis of many cancers, which originate from different types of tissue in the human body. Aberrant signals of FGF can promote tumor development by directly promoting the proliferation and survival of cancer cells, thus supporting tumor angiogenesis, redefining the tumor environment, and triggering metastasis [6].

Animal models have confirmed these hypotheses and show the oncogenic potential of bFGF signaling [7,8]. The involvement of FGFRs in different stages of tumorigenesis has allowed researchers to make them promising targets for FGFR-derived cancer therapies [9]. Thus, drugs acting against FGFRs could represent a prominent therapeutic approach for cancers. These types of drugs can exert direct and indirect anticancer effects [10].

Therefore, in light of these assumptions, we aimed to perform a comprehensive literature review, focusing on the bFGF biological axis and its cross-talk with other regulatory signaling cascades. In addition, the most current cancer therapies targeting the bFGF/FGFR axis, as well as its importance in the prognosis of cancer patients, were examined.

## 2. bFGF

Among the 23 currently identified FGFs, acidic FGF (FGF1) and bFGF were the first to be isolated in humans [11]. bFGF, also known as FGF-2, was initially localized in the brain and the pituitary gland [11]. bFGF is sequestered in the extracellular matrix (ECM) by heparin proteoglycans (HSPGs), which, in addition to protecting the protein from ECM protease hydrolysis, operate as co-receptors to promote bFGF-FGFR receptor bonds [12]. The affinity of bFGF-FGFRs is increased by the binding of bFGF to glycosaminoglycans present on the cell surface and the ECM [13]. bFGF binding induces receptor dimerization, thus conducting the establishment of a complex with at least two ligands, two receptors, and one HSPG [14].

bFGF as an extracellular mitogen is secreted from a wide range of cells, mainly smooth muscle cells and macrophages [15].

The mechanism of bFGF secretion can be defined as unconventional, and it is mediated by a type I mechanism characterized by direct protein translocation across the plasma membrane [16].

The molecular mechanism of this process is dependent on a small set of trans-acting factors linked to the plasma membrane. Indeed, bFGF membrane translocation is intermediated by the ability of bFGF to oligomerize and cross the plasma membrane in a PI(4,5)P2-dependent manner [17]. Membrane-inserted bFGF oligomers are dynamic translocation intermediates that are disassembled at the extracellular leaflet mediated by membrane proximal heparan sulfate proteoglycans [17]. Lastly, this process results in the exposure of bFGF on cell surfaces as part of its unconventional mechanism of secretion.

The activity of bFGF is exercised through binding with one of the four FGF receptors (FGFR1-4) present on the cell surface of different cell types, although most of its biological functions are attributable to the binding with FGFR1 [15].

Physiologically, bFGF has been recognized as an important factor in the development and function of numerous body organ systems.

bFGF has been extensively linked to the biological functions of the hematopoietic system; it has also been shown to be an important neurotrophic factor in the central nervous system (CNS) [18]. In a different manner, in the skin bFGF contributes to melanogenesis and the morphogenesis of suprabasal keratinocytes [18], while in the eye bFGF may be important in photoreceptor survival and may participate in photoreceptor signal transduction [18].

Moreover, a lesser-known feature of bFGF is its production as multiple isoforms via an alternative usage of translation start sites.

Five bFGF isoforms (18, 22, 22.5, 24, and 34 kDa) in humans and three bFGF isoforms in rodents (18, 21, and 23 kDa) and chickens (18.5, 20 and 21.5 kDa) have been identified [19]. These numerous isoforms are produced by an alternative initiation of translation on the bFGF mRNA [20]. bFGF isoforms have different subcellular localization and function, and can thereby have various impacts on the cellular microenvironment in a wide range of body districts [19]. Furthermore, the secreted forms of bFGF have been found to translocate into the cytosol and nucleus of their target cells after their interaction with the FGFRs present on the cell surface, thus regulating cellular processes in a paracrine/autocrine fashion [19].

These assumptions have been confirmed in several studies performed on neonatal cardiac myocytes, in which the upregulation of intracellular high molecular weight bFGF amplified cell proliferation while inducing bi-nucleation and changes in the nuclear morphology [21]. Likewise, in normal astrocytes, the translocation of bFGF from the cytosol to the nucleus was associated with proliferation, though contact inhibition between cells was linked with redistribution of the growth factor to the cytosol [19].

It has also been proved that intracellular bFGF contributes to several differentiation processes. Sherman et al., indicated that the intracellular high molecular weight isoforms of bFGF produced the trans-differentiation of avian neural crest-derived Schwann cell precursors into melanocytes [22].

In pathological conditions, bFGF is one of the most extensively studied peptides [23]; in this regard, numerous studies have highlighted the therapeutic potential of bFGF in wound healing, cardiovascular disease, and nervous system disorders [24,25,26]. The use of bFGF to protect neural stem cells laid the groundwork for the development of treatments for neurodegenerative diseases such as Alzheimer’s disease (AD) and Parkinson’s disease (PD) [27]. bFGF has also been studied as a therapeutic for brain repair after traumatic events, such as brain and spinal cord injuries [28,29], and has demonstrated prominent neuroprotective activity. Nevertheless, in CNS disorders, the therapeutic use of bFGF is limited by its short half-life in the blood and low blood–brain barrier permeability, as well as by its several side effects, such as a reduction in blood pressure [30,31].

As bFGF is a potent angiogenic factor, one of its main biological targets is the vascular system [32]. Preclinical reports have highlighted that bFGF improved myocardial perfusion and also provided cardioprotection during ischemia-reperfusion injury [33], while demonstrating a strong action against cardiac hypertrophy [34]. It has been proven that bFGF knockout (KO) hearts were protected from isoproterenol-induced cardiac hypertrophy [34]. Conversely, the hearts of bFGF transgenic mice showed an aggravated hypertrophic state [34]. In addition, clinical studies have demonstrated the efficacy of bFGF provision [35,36]. Recombinant bFGF has also been tested in peripheral artery disease and shown improvements in limb function [37,38]. Although some of the potential human health benefits of bFGF administration are known, its activity in different disease settings could prove harmful. In pathological cases, such as cancer, bFGF acts as a pivotal orchestrator in tumor-related genesis and growth, leading to poor outcomes for affected patients; thus, it represents an attractive target for antitumor therapy.

## 3. Function of bFGF in Tumor Microenvironment

**Figure 1:**
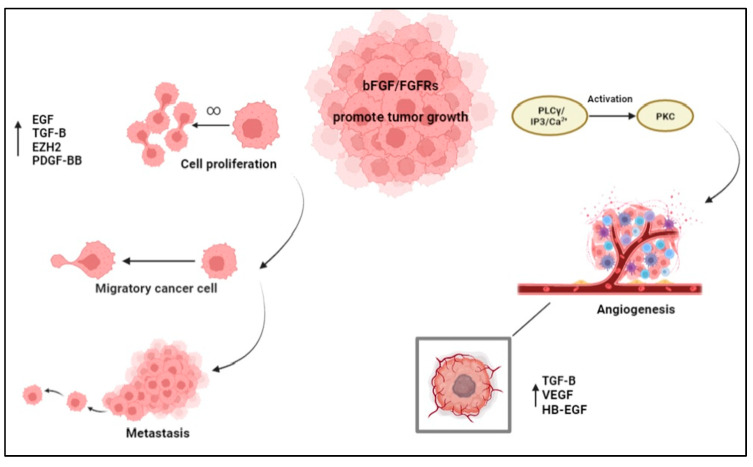
bFGF driving tumor evolution and spread. The figure presents the biological mechanisms controlled by bFGF, which contributes to both cancer cell growth and migration in other body districts, largely through neoangiogenesis.

Considering the ability of bFGF to regulate cell growth, angiogenesis, and other important biological processes, it clearly has an influence on the oncogenic course by controlling and redefining the tumor microenvironment, as illustrated in Figure 1.

Specifically, the control of the tumor environment exerted by the aberrant bFGF signaling pathway resulted in a significant increase in pro-tumorigenic mediators and in the downstream of tumor suppressor regulators at the same time. Overall, such reprogramming of the tumor mass due to bFGF hyperactivation notably increases the tumor growth rate by stimulating cell proliferation and tumor vascularization as well as decreasing the sensitivity to anticancer therapies.

Therefore, the understanding of the mechanisms underlying the bFGF signaling pathway could be useful in the development of different attractive biological targets, which could favor the control of the therapeutic response, thus improving the patients’ clinical outcomes.

### 3.1. bFGF and Angiogenesis

bFGF was discovered as a typical inducer of angiogenesis, and it has already been studied for three decades. Recent evidence indicates that bFGF plays different roles and controls signaling pathways, and it identifies bFGF’s typical role in angiogenesis [39]. bFGF represents a typically pro-angiogenic factor and is certainly the most distinguished in the large family of FGFs, being present in sites of chronic inflammation, tissue damage, etc. [40]. Interestingly, several studies have demonstrated the ability of bFGF to trigger neovascularization by using in vitro and in vivo models, particularly rodents or zebrafish species [41,42]. The action of bFGF on endothelial cells can be of the paracrine type; otherwise, it can also be produced endogenously by the endothelium, exerting its angiogenic activity through autocrine and intracrine modalities [40]. In medical conditions, including cancer, cooperation between angiogenesis and inflammation has been demonstrated [43]. In this regard, the bFGF-mediated angiogenic action can be induced by inflammatory states thanks to the action of the inflammatory cells and mediators that act on the endothelium by stimulating its release. bFGF contributes to the upregulation of other pro-angiogenic factors in the endothelium such as VEGF and angiopoietin-2 (Ang2) [44]. It is known that bFGF can also influence the metabolism of vascular endothelial cells by overregulating HK2, through which it monitors the glycolytic process and thus promotes angiogenesis [45]. Furthermore, it is important to underline the PLCγ/IP3/Ca^2+^ signaling as another canonical signaling pathway induced by FGF/FGFR, leading to the activation of PKC, an important regulating factor in angiogenesis [39].

Recently, a new study indicated that bFGF induces the expression of the splice factors SRSF1 and SRSF3 and the splice kinase SRPK1 in the HUVEC and HDMEC endothelial cell lines by modulating the splicing of the vascular endothelial growth factor 1 (VEGFR1) receptor towards splice variants known as soluble VEGFR1 (sVEGFR1), which are devoid of their transmembrane and intracellular segments [39]. These data pave the way for a new RNA splicing-dependent mechanism by which bFGF initiates blood vessel formation. Confirming this, a correlation was found between bFGF and sVEGFR1 in patients with squamous lung cancer [39]. Furthermore, since bFGF and VEGF promote angiogenesis by contributing to tumor growth and spread, they have been widely associated with shorter overall survival in non-small cell lung cancer patients [46], as well as in subjects affected by hepatocellular carcinoma and non-Hodgkin lymphoma [47,48].

Given the complex interplay of these factors in the tumor microenvironment, the development of successful anti-angiogenic therapies for cancer patients is a challenge for researchers.

### 3.2. Role of bFGF in Cell Proliferation and Metastasis

Cancer cells appear to secrete many FGFs, including bFGF, which induce excessive cell proliferation. For this reason, bFGF has been defined as a mitogenic agent, one which is mainly involved in the proliferation of numerous cells through interaction with FGFRs [49]. The crucial signaling in the bFGF-induced proliferation of different cell types is the Ras pathway [50]. Active Ras determines, in turn, the activation of a cascade of Ser/Thr Raf kinases as well as MEK and ERK1/2 [50]. However, the role of kinases such as Src, PKC, or P13-kinase appears to depend on the type of cell involved. Regarding bFGF activity, this growth factor would seem to contribute to cell proliferation both in situ and in metastases through angiogenesis and cell migration processes, respectively.

To confirm this, in vitro studies on original stem cells were conducted to identify the proliferative effects of bFGF [51].

For instance, in tumor cells, the overexpression of the enhancer of zeste homolog-2 (EZH2), which is associated with the polycomb-2 repressor complex (PRC2), promotes cell proliferation, cell migration, and invasiveness under the influence of bFGF [52]. Similarly, NDY1/KDM2B, a histone H3K36me2, H3K36me1, and H3K4me3 demethylase, is upregulated in the primary tumor environment driven by bFGF signals [53]. Moreover, in the tumor context, bFGF, in cooperation with another important angiogenic factor such as platelet-derived growth factor-BB (PDGF-BB), promotes metastasis through angiogenesis and vasculogenesis courses [54]. As described above, bFGF triggers cell proliferation, modulating the EZH2 pathway, a critical component of PRC2; this mechanism largely contributes to the progression and metastasis of different tumoral forms [55]. Accordingly, Chengye and colleagues, in a study on hepatocellular carcinoma (HCC), demonstrated the ability of bFGF to spread tumor metastasis by the interaction with its receptors, thus promoting and regulating cell motility [56].

### 3.3. bFGF and Chemoresistance

One of the main causes of cancer treatment failure is represented by the development of resistance due to the effects of drugs; this is known as chemoresistance.

Chemoresistance causes disease relapse, leading to the onset of metastases; it represents the main obstacle to cancer therapy and a difficult clinical challenge to overcome in improving cancer patients’ outcomes. Therefore, it is very important to understand its molecular mechanisms and to discover novel therapeutic approaches to cancer therapy. In this complex event, several molecular mechanisms have been recognized as major players in chemoresistance, including transporter pumps, oncogenes, tumor suppressor genes, mitochondrial alteration, DNA repair, autophagy, epithelial-mesenchymal transition (EMT), cancer stemness, and exosomes [57]. In particular, it would seem that it is not just one factor that plays a key role in drug resistance but that many biological cross-talk factors contribute to its establishment.

For instance, the encoding proteins of oncogenes (EGFR-Akt-NF-κB) might modulate the expression of the apoptosis-related genes and contribute to EMT, cell stemness, and autophagy during chemoresistance [57]. Moreover, the dysregulation of FGF/FGFR signaling was associated with enhanced chemotherapy resistance and, consequently, with poor clinical outcomes. Indeed, fibroblasts, which are abundant in the stroma of carcinomas at advanced stages of tumor diseases, can mediate resistance to treatment via bFGF secretion [58]. These biological mechanisms involve a network of biological interplays, through which bFGF exerts its action and promotes the resistance of cancer cells to conventional chemotherapeutics.

In this regard, it was demonstrated that bFGF promoted chemoresistance by deregulation of JAK/STAT signaling in the malignant Hodgkin and Reed–Sternberg cells of relapsed and refractory Hodgkin lymphoma patients [59].

Other evidence has proved that ASK1 is required for stress-mediated apoptosis in ECs and that bFGF upregulation supported the formation of a complex between Raf-1 and ASK1 at the mitochondria, suppressing ASK1 activity and providing chemoresistance to endothelial cells [60].

Moreover, resistance to cisplatin and fludarabine was related to high bFGF concentrations, as revealed by examinations in patient plasma or urine [61].

Song and colleagues probed the bFGF-induced resistance, highlighting an extracellular mechanism which was reversible by treatment with neutralizing mAb and suramin, suggesting a process that involves the binding of this growth factor to its receptor [61].

Overall, these scientific findings demonstrated the epigenetic mechanism by which cancer cells use the unique microenvironment of solid tumors and metastases to elude cytotoxic insult, and they establish an important role of extracellular bFGF in tumor sensitivity to chemotherapy.

## 4. bFGF and Cancer

As stated, the bond between bFGF and FGFRs leads to the auto-phosphorylation of intracellular tyrosine residues, which consequently instigates tumor cell proliferation and invasion [32]. This bFGF capacity results in the upregulation of several proteins, such as MMP-1, HGF, Bcl2, Survivin, MMP-9, and MMP-13, raising the invasive and anti-apoptotic properties of tumor cells [62]. Thus, the deregulation of bFGF signaling has been described in various tumor types.

### 4.1. bFGF in Brain Tumors

Several studies reported the importance of bFGF signaling in the brain. Sie et al., demonstrated that the pediatric low-grade astrocytoma cell line exhibited high rates of FGFR1 expression (34–51%) compared to isotypic controls [63]. Further confirmation of this study is given by Trisolini et al., who reported in their study the frequent mutations of FGFR1 in pilocytic astrocytomas of the optic pathway, proposing this receptor as a possible therapeutic target in the oncological field [64]. bFGF is an important oncogenic factor in glioblastoma, contributing to vascularization, angiogenesis, and glioma growth cells [65]. The secretion of bFGF by the glioblastoma cells contributes to the increase in chemotherapy resistance as it improves the function of the endothelial cells of the blood–brain barrier (BBB) [66]. A study conducted on C6 glioma cells further confirmed the involvement of bFGF in the progression, as well as the aggressiveness and invasiveness of the glioblastoma. In particular, it has been found that bFGF, together with epithelial growth (EGF), is responsible for the potential self-renewal of glioblastoma stem cells (GSC). Indeed, the removal of bFGF from glioma stem cell lines determines their differentiation, which was not observed when the cells were in the presence of bFGF [67].

In the context of angiogenesis, new probable angiogenic inhibitors have been identified, such as NSC12, which are analogues of thrombospondin and capable of directly interfering with the binding between bFGF/FGFRs, reducing cell proliferation and modulating the angiogenetic process controlled by bFGF. This study was conducted both in vitro and in primary WBC cells from patients [68]; in addition, an in vivo model was used to test the antitumor activity of NSC12 [68].

The inhibition of FGFs could represent a promising therapeutic approach to counteracting tumor progression, given the involvement of these factors in tumorigenesis. The idea of obtaining molecules capable of inhibiting the activity of FGFRs led to the development of Fisogatinib and Futibatinib, two effective and safe compounds which are capable of improving the neoplastic condition [69]. Fisogatinib, a quinazoline derivative, exerts its antitumor action by specific binding to the FGFR4 receptor subtype. Clinical studies report the activity efficacy of Fisogatinib [70,71]. Regarding its mechanism of action, Fisogatinib covalently binds a unique cysteine residue found in FGFR4 (Cys 552), conferring a high selectivity towards FGFR4 over other FGFRs [70].

Futibatinib, on the other hand, is a pyrazole [3,4-d] pyrimidine derivative, which irreversibly and selectively inhibits FGFR receptors [72]. This action consequently determines the inhibition of the signal transduction pathway mediated by FGFRs and therefore of the proliferation of tumor cells, thus enhancing its antitumor activity. Phase I/II clinical trials of Futibatinib are ongoing in patients with advanced metastatic solid tumors presenting with FGFR aberrations [73]. In vitro and in vivo studies were conducted by Sootome et al.; the studies demonstrated the antiproliferative activity of Futibatinib by covalent binding to the FGFR kinase domain [72]. These assumptions were further validated in primary CNS tumors by Meric-Bernstam and colleagues [74], as well as by Khabibov et al. [75], who propose, respectively, Futibatinib and Fisogatinib as potential therapeutic strategies for CNS tumors.

Furthermore, elevated bFGF plasma levels in brain tumor patients were correlated with a high mortality rate and poor survival, thus advancing the development of pharmacological inhibitors capable of directly interfering with the bFGF/FGFR signaling axis as a valid approach for brain tumor therapy [76]; however, further in-depth clinical studies are needed to clarify both the prognostic and the therapeutic value of bFGF in brain tumors.

### 4.2. bFGF in Gastrointestinal Cancers

Preclinical investigations validated bFGF as an oncogenic factor in esophageal squamous cell carcinoma (ESCC), demonstrating that its downregulation in the ECA109 cell line inhibited cell proliferation, migration, and invasion [77]. In addition, Shi and colleagues suggested LY294002, a PI3K inhibitor, as a therapeutic strategy for alleviating the tumorigenic effects of bFGF [77]. In a clinical study involving 96 ESCC patients, Li et al., correlated bFGF with the depth of tumor invasion and lymph node metastasis, confirming its role in angiogenesis and cancer progression [78]. Esophageal adenocarcinoma (EAC) is a highly lethal cancer whose development and progression seem to be supported by abnormal levels of bFGF and VEGF [79,80]. In fact, in human specimens, the expression of bFGF was significantly increased in EAC compared with normal squamous mucosa [80]. Overall, the prominent presence of bFGF is negatively correlated with ESCC and EAC patients’ outcomes, increasing the risk of relapses as well as reducing overall survival post-surgical resection [81,82].

Numerous studies have proved the involvement of bFGF in human gastric cancer (GC) by demonstrating higher bFGF concentrations in GC patients than in control groups [83]. Because of that, in the last decade one of the main goals of scientific research has been to identify effective targeted treatments. Recombinant human endostatin was able to limit the proliferation of gastric cancer cells, inducing apoptosis in a GC murine xenograft model [84]. Peptidomimetics such as P29 and P32 exerted good antitumor activity, suppressing the bFGF-induced proliferation of GC cells while inhibiting the activation of AKT and Erk1/2 cascades [85,86]. Similarly, microRNAs such as miR-381-3p and miR-195 directly targeted bFGF targets, powerfully decreasing the tumorigenesis of GC; thus both represent potential therapeutics for affected patients [87,88]. In another paper, Guo et al., probed the biological activity of toxicarioside A in reducing cell viability, cell growth, and cell migration and invasion in SGC-7901 GC cells through the modulation of NF-κB/bFGF/FGFR1 signaling [89]. bFGF also regulates immune infiltration, being directly linked to a higher extent of M2 macrophage intrusion in a clinical study enrolling a total of 726 GC patients [90]. Maspin, known as Serpin peptidase inhibitor, decreases the cell invasion and migration of GC by influencing the ITGB1/FAK pathway, thus restraining the EMT and angiogenesis processes [91]. Regarding the prognostic value of bFGF in GC, we found discordant opinions. Indeed, if on one hand bFGF was considered an unfavorable prognostic factor in GC [90,92], different evaluations advise only VEGF, not bFGF, as a reliable marker for assessing tumor progression and, in particular, clinicopathological factors or survival [93].

bFGF is also one of the most described growth factors in colorectal cancers (CRC) [94]. A large body of evidence aimed to evaluate the bFGF role in CRC; this is proven by the considerable amount of assessment of the potential treatments and the discoveries of its biological cross-talk, e.g., with K-RAS signaling [95,96]. For instance, Catalpol, an iridoid glucoside with anti-inflammatory activity, blocked the cell proliferation, growth, and invasion of CT26 lines by controlling inflammation and angiogenesis in the tumor microenvironment [97]. Similarly, Oridonin administration in the xenograft model of human colon cancer influences both JAK2/STAT3 signaling and angiogenesis-related factors, including bFGF, exhibiting a worthy anticancer effect [98]. Among other natural compounds, morin and pomegranate have also been shown to decrease bFGF levels in the colons of rats and colon (colo205) cell lines, respectively [99,100]. Moreover, as disclosed by Samie and colleagues, ascorbic acid treatment substantially reduced the expression of bFGF, bFGFR, PDGF, PDGFR, and PLC- γ in the HT29 cell line [101]. The low survival rate of CRC patients is principally due to the chemotherapeutic resistance of tumor cells; relatedly, it has been reported that bFGF could regulate the epigenetic mechanisms of drug resistance, thus representing a novel potential target for improving the sensitivity of tumor cells to chemotherapeutic agents [102]. According to preclinical findings, clinical studies confirmed bFGF as a clinicopathological factor and highlighted its possible role as a marker of metastatic progression and patient prognosis [103,104].

### 4.3. bFGF in Liver Cancer

Hepatocellular carcinoma (HCC) is the most common form of liver cancer in adults, and its incidence and mortality rate are increasing worldwide [105,106]. Extensive data show that the FGF/FGFR signaling axis participates in contributing mechanisms to HCC development. CT perfusion-related parameters and serum bFGF levels in patients with primary liver cancer are abnormally expressed, and there is a strong correlation between the two, which might aid clinical diagnosis and treatment [107]. Liu et al., reported that the upregulation of bFGF and ACLY by ONECUT2 promotes the formation of HCC metastases in mice [108]. Likewise, in the human hepatoma cell line SMMC-7721 and Hep3B, the angiogenesis pathways such as PI3K-Akt, VEGF/KDR, and Angiopoietins/Tie2 and the angiogenic factors such as bFGF [109] were upregulated and consequently suppressed by Plumbagin administration [110].

In humans, Sorafenib-treated HCC patients were exposed to evidently lower levels of VEGF, bFGF, and AFP in serum, resulting in prolonged survival [111]. Changes in the circulating angiogenic factors in HCC were also reported by Joo and colleagues. In a total of 240 patients, comprising 156 HCC, 37 cirrhosis, and 47 chronic hepatitis subjects, the bFGF levels revealed a considerable change [112].

A similar trend was also detected in patients with hepatitis B virus-associated HCC [113] and primary liver cancer [107], suggesting that bFGF has importance in clinical diagnosis and treatment.

For this purpose, combined therapies of transcatheter arterial chemoembolization (TACE) with thalidomide have been successfully used in downregulating VEGF and bFGF levels. In addition to this, the obtained data also indicated an improvement in patients’ overall survival after the 3-year follow-up [114]; this strongly associated the shorter survival times and higher recurrence rates with bFGF [115,116].

### 4.4. bFGF in Pancreatic Cancer

Tumors of the pancreas can originate in both the endocrine and the exocrine portions of the pancreatic gland [117]. Pro-angiogenic factors such as VEGF and bFGF largely contribute to the proliferation, invasion, and migration of pancreatic cancer cells [118]. In contrast, Quercetin-3-O-glucoside and Celecoxib acted as bFGF inhibitors, avoiding the expansion of local metastasis induced by various growth factors in pancreatic cancers and promoting their use as an effective adjuvant to boost chemotherapeutic agents [119,120]. Moreover, Chen et al., elucidated the interplay between the heparanase (HPA)/syndecan-1 (SDC1) axis and bFGF, suggesting HPA/SDC1 silencing as a valuable therapeutic strategy in decreasing bFGF [121]. Clinically, a case-control study, including 34 PDAC patients, shed light on the potential role of bFGF and PlGF as diagnostic biomarkers [122], thus supporting bFGF as a valuable candidate for anti-angiogenic treatments [123].

Moreover, the overexpression of bFGF in pancreatic cancer tissue was associated with poor prognosis, as indicated by Ghaneh et al. [124], thus evidencing the role of this growth factor in significant correlations with survival and/or treatment responsiveness in pancreatic cancer patients.

### 4.5. bFGF in Oral Cavity

Oral tongue squamous cell carcinoma (OTSCC) is one of the most prevalent tumors of the head and neck region [125]; bFGF emerged as one of the most important contributors to its progression. In the HSC3 cell line, anti-VEGF or anti-bFGF antibodies, either alone or in combination, were effective in decreasing the activity of the urokinase-type plasminogen activator [126]. Foorotan et al., investigated the association between bFGF, the receptors for bFGF, and neo-angiogenesis in 51 OTSCC patients, of whom 26 presented metastasis in cervical lymph nodes [127]. The OTSCC tissues revealed a considerable bFGF expression in tumor-infiltrating lymphocytes. However, their data indicated a non-relationship between bFGF and neoangiogenesis in lingual carcinomas or nodal metastases [127]. In addition, more recent studies have sought to clarify the role of bFGF and its prognostic value in the oral cancer framework. Mariz et al., through a recent large-scale study, highlighted how bFGF positivity in the stroma was connected to vascular invasion and worse patient prognosis [128], thus confirming the pivotal function of bFGF in predicting adverse outcomes for OTSCC patients.

In a similar manner, studies performed in the last three years have pointed out the relevant alterations in FGF signaling in head and neck squamous cell carcinoma (HNSCC). For instance, a preclinical study with TAS102-treated cells and a clinical study with HNSCC patients [129,130] highlighted FGF signaling alterations as relevant drivers of tumorigenesis. Confirming this, preclinical assessments have indicated curcumin as a potential natural compound to inhibit 4NQO-induced tumorigenesis via modulation of the bFGF/FGFR-2 axis [131] and AZD4547 as a new chemical molecule targeting the FGFR/Akt/SOX2 axis to overcome paclitaxel resistance in HNSCC [132]. Furthermore, alterations in angiogenesis-modulating genes can influence therapy outcomes and tumor progression. Specifically, bFGF rs1048201 CC homozygotes presented a higher risk of death and care failure, thus leading to poor prognosis in HNSCC patients [133].

### 4.6. bFGF in Lung Cancer

Many studies have examined the relationship between human bFGF overexpression and survival in lung cancer patients, but the results have been conflicting [23]. However, although the role of the FGF/FGFR axis in lung cancer development and progression is not yet clear, several therapies have demonstrated that the downregulation of bFGF expression leads to positive outcomes.

To counteract tumor progression, a murine monoclonal antibody (mAb), E12, was produced against human bFGF, targeting the binding site of bFGF with FGFR1. Specifically, mAb-E12 inhibited Lewis’s lung cancer (CLL) metastasis by increasing E-cadherin expression via the transcriptional repressor 1 (snail) pathway of the AKT GSK3 β snail family, suggesting that mAb-E12 could be a promising anticancer antibody [134]. A more in-depth study on the immune landscape of lung cancer, including the ability to evade the immune response, has led researchers to investigate further possible molecular targets capable of revolutionizing therapeutic advances [135]. In this regard, studies in the literature report that bFGF and FGFR1 are associated with drug resistance in lung cancer [134]. Following gene expression screening in lung tumor tissue, especially in NSCLC, it has been shown that both bFGF and FGFR1 and FGFR2 and epidermal growth factor (EGFR) show elevated levels of expression, which thus associates them with an increase in chemotherapy resistance [136]. Compared to EGFR, however, bFGF and FGFR1/2 report few mutations, which therefore makes them potential reliable targets for lung cancer therapy [137]. In the first stage of tumorigenesis, junctions exist between tumor cells and normal epithelial tissues that prevent tumor cells from forming complete tissue. Following the development of cancer, some molecules of the surface adhesion of the tumor cells are altered; consequently, normal junctions are lost between the cells. This process sees the involvement of an important adhesion molecule, E-Cadherin [138]. Furthermore, bFGF has been reported to collaborate with other factors to strengthen the involvement of the bFGF/FGFR signaling pathway [139]. For example, during the EMT process, the transformation of growth factor-β induced the switching of the FGFR isoform, causing the cells to become sensitive to bFGF [140]. Similarly, the FGF-phosphoinositide 3-kinase-protein kinase B (AKT)-glycogen synthase kinase 3β (GSK3β) signaling pathway regulates EMT and increases the invasiveness of tumor cells [141]. The bFGF/FGF signaling pathway promotes EMT by the downregulation of E-Cadherin expression [142]. A study conducted by Behrens, C. et al., showed how bFGF/FGFR1-2 signaling plays an important role in the pathogenesis of squamous carcinoma cells and adenocarcinoma; the study considers the immunohistochemical expression of the three molecules in 426 epithelial samples containing histologically normal, hyperplastic, squamous metaplastic, or squamous dysplastic bronchial epithelia adjacent to NSCLC obtained from 130 patients [143]. Overall, NSCLC tumor cells demonstrated higher levels of bFGF and FGFR1-2 protein expression than the histologically normal bronchial epithelium. Squamous dysplastic lesions showed significantly higher levels of expression than squamous metaplastic lesions for all three markers [143]. In adenocarcinoma specimens, Behrens, C. et al., found differences in the expression of the three markers between smokers and non-smokers. In particular, they obtained a higher cytoplasmic expression of FGFR1 in smokers, while FGFR1/2 was more expressed at the nuclear level in non-smokers. Therefore, based on the above and the results obtained from this study, it is clear that bFGF/FGFR1-2 signaling represents a promising target for lung cancer therapy [143], although it has not yet been fully investigated.

Regarding the prognostic value, a meta-analysis showed that bFGF overexpression is a potential indicator of worse prognosis for patients with operable non-small cell lung cancer (NSCLC) and small cell lung cancer (SCLC) but is not associated with outcome in advanced NSCLC, suggesting that high bFGF expression is highly related to poor prognosis [23].

### 4.7. bFGF in Kidney Cancer

Few, but accurate, studies have investigated the role of bFGF in clear cell renal cell carcinoma (ccRCC) tissues and human cell lines. In particular, in the field of ccRCC, several miRNAs have been proposed as tumor suppressors [144,145]. Zhang et al., demonstrated the beneficial effects of miR-148b-3p in stimulating renal carcinoma cell apoptosis while suppressing cell proliferation and migration growth via the FGF2-FGFR2 pathway [144]. Likewise, miR-203 post-transcriptionally targets bFGF, thus avoiding renal cancer cell growth and metastasis rise [145]. In the RCC xenograft murine models employed by Matsuki and colleagues, a combination of Lenvatinib and Everolimus proved to be a powerful anti-angiogenic treatment against FGF-activated endothelial cells [146]. From the clinical side, the prognostic value of bFGF was evaluated in the serum of 74 RCC patients; through this evaluation, it was discovered that metastasized patients revealed higher bFGF expression and lower long-term survival [147]; also highlighted was the correlation of its high expression with tumor growth and unfavorable prognosis [40].

### 4.8. bFGF in Bone Tumors

The effects of FGF/FGFR signaling in osteogenesis are complex and not yet fully understood in bone biology [148]. These receptors, once activated, lead to phosphorylation of the FGFR 2a substrate, phospholipase recruitment Cγ (PLCγ), activating cascades, and downstream networks (e.g., mitogen-activated protein kinase (MAPK) and phosphatidylinositol-3-kinase (PI3K)/protein kinase B (AKT) but also the signal transducer and transcription activator (STAT) [137]). The FGF/FGFR signaling pathway cooperates with other pathways involved in osteogeneses, such as bone morphogenetic proteins (BMPs) and the Wnt ligand, glycoproteins which are rich in cysteine and highly hydrophobic, especially in the canonical pathway. Both in vitro and in vivo studies report how FGFs improve canonical BMP2 signaling by promoting the nuclear accumulation of β-Cadherin in osteoblasts, thus regulating the fate and differentiation of mesenchymal stem cells [149]. Furthermore, it has also been shown that bFGF is necessary for the positive effects of the parathyroid hormone (PTH) on osteoblast proliferation and differentiation, which in turn stimulate FGFR1 and FGFR2 in osteoblasts [149]. Therefore, the bFGF/FGFR axis controls bone remodeling; it also regulates the activation and function of osteoclasts. bFGF determines the proliferation of osteoclast precursors and thus stimulates bone resorption through the activation of FGFR1 and MAPK [150]. Cellular and genetically modified mice (GEM) studies conducted on bFGF have determined that different isoforms of bFGF [148], of both high and low molecular weight, appear to have opposite effects on bone mass. Low molecular weight bFGF determines an increase in bone mass in mice by modulating the Wnt/β-Cadherin signaling pathway [151]. Conversely, high molecular weight bFGF is a negative regulator of osteoblast differentiation and matrix mineralization [152]. In the last stage of cancer development, metastasis, the involvement of the FGF/FGFR axis was found, in which mutations in the receptors and/or the overexpression of their ligands and/or receptors were reported. Finally, since bFGF modulates angiogenesis and osteogenesis, two related processes in bone formation, it was thought to be useful as a molecular target in tumors involving bone [153]. Tsubaki, M. et al., were the first to conduct studies on the inhibition of the growth factors bFGF, HGF, and TGF- β in osteosarcoma cells [154]. Statins have been reported to inhibit the expression of bFGF, HGF, and TGF-β in osteosarcoma cells through the inhibition of geranylgeranyl pyrophosphate (GGPP) biosynthesis in the mevalonate pathway. In particular, statins inhibit the membrane localization of K-Ras and Rho, two important oncogenes that control both cellular processes and signaling pathways in eukaryotic cells; they also suppress the phosphorylation of ERK1/2 and Akt [155]. Studies in the literature suggest that ERK1/2 and Akt activation correlates with bFGF expression [156]. In one study, the expression of bFGF was inhibited by the suppression of ERK1/2 and Akt, which consequently restrained the synthesis and secretion of HGF in the MG-63 osteosarcoma cell line [157]. Furthermore, Tsubaki, M. et al., demonstrated that inhibition of ERK1/2 and Akt inhibits the expression of angiogenic factors in osteosarcoma cells, which thus suggests the use of statins as potential anti-angiogenic agents for the treatment of osteosarcoma [154]. Therefore, considering the involvement of the ERK1/2 and MAPK signaling pathways, their blocking contributes to the reduction in the proliferative activity of bFGF against osteoblasts; thus, they encourage the development of a further therapeutic approach.

Therefore, considering the involvement of bFGF in the invasiveness of bone tumors, through activation of the ERK1/2 and MAPK signaling pathways, it was considered appropriate to act by blocking this axis to reduce the morbidity and mortality associated with bone cancer metastases; these assumptions were also confirmed by clinical studies in which high serum levels of bFGF were correlated with poor overall survival [104].

### 4.9. bFGF in Thyroid Cancer

Recent scientific evidence has shown that angiogenic factor profiling can be useful in the deepening of tumor biological tools and highly useful in strengthening therapeutic strategies for papillary thyroid carcinoma (PTC) [158]. PTC patients revealed substantial bFGF levels when compared to healthy controls; however, a total thyroidectomy surgical procedure resulted in the successful decreasing of bFGF overexpression [158]. Moreover, miRNAs such as miR-27b-3p and miR-195 were probed in the context of thyroid cancer [159,160]. First, the involvement of miR-27b-3p in human anaplastic thyroid cancer (ATC), particularly in the Doxorubicin resistance of ATC cells, was validated [159]. Thus, Xu and colleagues recommend a targeted inhibition of miR-27b-3p to overcome chemoresistance in ATC patients [159]. In addition, Yin et al., highlighted the biological function of miR-195 in papillary thyroid carcinoma (PTC), whose overexpression limits proliferation, migration, and invasion in PTC cell lines, as well as bFGF levels [160].

Through in vitro and in vivo studies, it has been shown that the P9 peptide acts as an effective bFGF/FGFR antagonist, while the P11 peptide inhibits the PI3K/Akt/mTOR signaling pathway [161]. Comparably, in follicular thyroid cancer (FTC), the overexpression of Prospero homeobox 1 (PROX1), a key regulator of lymphangiogenesis, resulted in a decrease in several FGF signaling pathway members, including the downregulation of bFGF [162]. In clinical analyses, bFGF resulted in being a valuable marker in the distinction between invasive and non-invasive PTC, as well as between benign or malignant specimens [163]. Therefore, from this perspective, the evaluation of bFGF expression in the postoperative phase would be useful both as a discriminating factor by type of PTC and in the further evaluation of the risk of recurrence; thus, it would provide helpful assistance in the diagnosis and identification of thyroid cancer [163].

Therefore, from this perspective, bFGF could prove to be an optimal prognostic factor for the evaluation of the biological behavior and risk of recurrence in the postoperative phase, and thus, it could be an aid in the diagnosis and identification of thyroid cancer [163].

### 4.10. bFGF in Bladder Cancer

In bladder cancer, bFGF plays an important role in controlling angiogenesis, contributing to tumorigenicity and subsequent metastasis through interaction with its receptors [164]. Furthermore, the development of early relapses has been reported to result in the increased expression of bFGF messenger RNA (mRNA) in superficial bladder cancer [165]. In this regard, in cystectomy, bladder cancer samples were subjected to neoadjuvant chemotherapy, resulting in the high expression of bFGF being an independent prognostic factor for disease recurrence [166]. Therefore, these results suggest a deeper clarification regarding bFGF expression in chemoresistant bladder cancer cells, particularly with regard to its role in bladder cancer recurrence after intravesical chemotherapy [167]. Chen, Y. et al., conducted a preliminary investigation to better investigate the role of bFGF in the recurrence of chemotherapy-resistant cancer [167]. The results highlighted an increased bFGF density in recurrent bladder cancer tissues compared to primary tumor tissues. To confirm this, an increase in bFGF expression was observed in an in vitro study conducted on chemoresistant 253J/DOX cells [167]. Furthermore, it is surprising to note that bFGF expression in surviving cells after treatment with different chemotherapy drugs is upregulated, indicating that this could be a universal phenomenon after chemotherapy [168]. The increase in bFGF in chemoresistant cancer cells favors the interaction of cancer endothelial cells, which helps to accelerate tumor recurrence. Therefore, considering the role of bFGF in tumorigenesis, Chen, Y. et al., evaluated antagonism against bFGF using the neutralizing antibody, which, however, did not affect the growth of 253J/DOX cells [167]. This study led to the conclusion that the upregulated expression of bFGF represents an important mediator of angiogenesis in residual bladder cancer after intravesical chemotherapy [167]. Furthermore, a study conducted by Ramy F. Y. and colleagues confirmed the overexpression of bFGF in bladder cancer cells, thus associating it with the aggressiveness and relapse of this tumor and thus suggesting the prognostic role of bFGF in bladder cancer [169]. Tibor S. and colleagues conducted a clinical study analyzing serum samples from 82 bladder cancer patients, comparing them with 20 healthy patients, with the aim of better understanding bFGF in bladder cancer and assessing the prognostic significance of their serum levels [170]. Interestingly, of the different receptor subtypes FGFR3 is the most involved in the progression of bladder cancer [171]. The isoforms FGFR3b and FGFR3c have been identified in some bladder cancer cells, which bind a wide range of FGFs, including bFGF, indicating autocrine or paracrine FGFR3 signaling in some bladder tumors [172]. Moreover, a strong association of FGFR3 mutation with low tumor grade and stage mutation has also been identified with high frequency in urothelial papilloma [173], a proposed precursor lesion for low-grade papillary urothelial cancer [174].

In this regard, the urine of bladder cancer patients was analyzed and several FGFs were identified, including bFGF [175]; its high presence in cancer patients may be derived from the basement membrane freeing itself during tumor invasion, where it stimulates angiogenesis at the invasion margin [176]. To date, the therapeutic approach for the treatment of bladder cancer consists of combined interventions of surgery, chemotherapy, radiotherapy, and/or immunotherapy. Although surgical resection is the main therapeutic approach, intravesical treatment with the bacillus Calmette-Guerin (BCG) [177], used in the past to vaccinate against tuberculosis, has been remarkably effective in reducing the neoplastic lesion [178]. Doxorubicin also seems to be able to reduce the frequency of recurrences; more than half, about 55–75% of cases, developed the tumor again, with muscle invasion after primary treatment [179]. Unfortunately, the use of these conventional chemotherapeutic drugs may induce chemoresistance through the upregulation of ATP-binding cassette proteins or the activation of anti-apoptotic signals [165,180]. Furthermore, the results obtained from the various studies propose the inhibition of FGFR3 as a further therapeutic approach, given its high involvement in tumor development, through intravesical treatment, thus offering the possibility of the complete elimination of tumor cells after surgical resection, preventing recurrence [181]. If this strategy were to be validated in humans, it would consequently reduce the need for long-term monitoring while also reducing the associated high costs and patient morbidity.

From a clinical point of view, in-depth studies are needed regarding the involvement of the bFGF/FGFR signaling axis in bladder cancer; this growth factor may represent an important biomarker for diagnostic purposes and contribute to the development of new therapeutic strategies. As a prognosticator, elevated bFGF concentrations were detected in samples of bladder cancer patients compared to controls, suggesting that this growth factor could be a reliable marker in the risk stratifications of bladder cancer patients [170].

### 4.11. bFGF in Prostate Cancer

Many FGFs are involved in the development and progression of prostate cancer. In this regard, several studies have been conducted, in vitro and in vivo, which have reported a controversial role of bFGF in prostate cancer with regard to both its cellular localization and the clinicopathological phenotype and prognosis. In the past, bFGF studies have been conducted using both the PC3 and DU145 cell lines and by synthesizing it from human prostate stromal cell cultures, where its presence was detected not only in cells in the normal state but even in a hyperplastic and malignant state [182]. Further confirmation of this study was obtained from the research of Sinowatz, F. et al. [183], in which bFGF expression was found in stromal cells in both benign prostatic hyperplasia and prostate cancer [184] from the studies of immunohistochemistry and molecular biology. In this context, Pecqueux et al., demonstrated the correlation existing between the high expression of bFGF in the tumor stroma and a high rate of postoperative relapse [185]; moreover, exogenous bFGF is capable of modulating genomic instability, thus promoting the progression of prostate cancer by increasing DNA damage [186]. Conversely, some studies have reported the expression of bFGF in the tumor epithelium by immunohistochemistry [187]. Among the different receptor subtypes of bFGF, FGFR1 is the receptor involved in angiogenesis [188]. To confirm what has been reported, a murine study was conducted, using the FGF/FGFR complex in prostate cancer, in which the high epithelial and mesenchymal interaction was reported [68], although these interactions are necessary for normal prostate organogenesis [189]. Considering the involvement of bFGF in the progression of prostate cancer, it is possible to neutralize the activation of the bFGF/FGFR axis, modulating their interaction at an extracellular and intracellular level.

The first approach to this strategy is represented by the ability to inhibit the production of angiogenic growth factors [190], particularly by stopping the production of FGF, through the use of chemotherapeutic agents such as taxane and docetaxel, which both downregulate bFGF expression [191]. Thanks to advances in the research, it has been possible to develop new strategies for the treatment of prostate cancer. In the case of elderly patients or those with other serious illnesses, it is possible to not implement any type of therapy until symptoms appear [192]. When the tumor is of a low risk, it is possible to not proceed with any treatment but to initially carry out periodic checks (PSA, rectal examination and biopsy), in order to monitor the progress of the tumor [193]. To enhance the anti-angiogenic and antitumor activity, an antisense bFGF oligonucleotide was produced which was capable of blocking the production of FGF by both tumor and endothelial cells [194]. Furthermore, another strategy involved the production of various second messenger inhibitors involved in the expression of FGFs, such as PKC, JAK, PI-3K, c-jun, ERK, JNK, STAT1, and STAT3 [195]. Therefore, as indicated above, the bFGF/FGFR axis could represent an important anti-angiogenic target for the treatment of prostate cancer.

Therefore, considering the poor prognosis associated with the elevated serum levels of bFGF in patients with prostate cancer, the use of combinational therapies could have a positive outcome for individuals resistant to traditional therapies [196].

### 4.12. bFGF in Breast Cancer

Breast cancer affects the cells of the mammary gland, which multiply uncontrollably, turning into malignant cells [197]. FGF/FGFR signaling can elicit an oncogenic action [198], although some studies report that the FGF/FGFR axis can exert an antitumor action in certain contexts [199]. A cohort study of 391 patients with breast cancer reported FGF and/or FGFR aberrations of approximately 32%; these aberrations contributed to tumor progression [200].

Fibroblast cells, MCF7, T47D, MDA-MB-231, BJ, and IMR90, were used to study the involvement of ECM in estrogen signaling by interacting with bFGF in ER-positive breast cancer cells via the signaling pathway of Erk1/2 [201,202]. These obtained results confirm that bFGF reduces the sensitivity of ER-positive breast cancer cells to endocrine therapies in vitro [203]. Moreover, considering that bFGF is linked to ECM, the use of the specific inhibitor of bFGF, PD170374, was able to stop the ECM/FGF-mediated regulation of ER. Therefore, from that which has been reported, the FGF/FGFR system can be considered a promising therapeutic target to establish more complete anticancer strategies.

Moreover, limited, but relevant, clinical evidence has elucidated bFGF as a useful prognostic parameter to establish the tumor stage in women with breast cancer [204]. These findings indicate that bFGF is prognostically relevant in pathological features of breast cancer, such as tumor stage, grading, nodal stage, and survival, and suggest that it is a novel parameter for worse prognosis in nodal-negative breast cancer patients [204].

### 4.13. bFGF in Ovarian and Cervical Cancer

Along with breast cancer, ovarian and cervical cancer are tumors that most commonly cause death in young adult women [205]. Numerous factors are involved in the promotion of cervical cancer, including bFGF. Indeed, the scientific evidence reports a strong expression of bFGF and FGFR2; in particular, the bFGF/FGFR signaling axis contributes to the development of invasive cervical cancer [206].

An in vitro study conducted on human cervical cancer cell lines, HeLa, SiHa, and CaSki, further confirmed the role of the bFGF/FGFR pathway, in which increased bFGF was reported [207].

The release of bFGF by these cells can be inhibited by treatment with specific inhibitors for FGFR, whereas the gene alteration of FGFR and bFGF has been found in patients with uterine cancer. It is interesting to note that, through the use of confocal microscopy and subcellular fractionation, the presence of FGFR1 and FGFR2 was found in the nucleus [208].

Therefore, treatment with the FGFR inhibitor, PD173074 [209], and/or the application of RNAi targeted at FGFR1/2 reduces the expression of the oncoprotein HPV 16/18 E7, which is probably the main culprit in malignant transformation and cervical cancer development, indicating, in turn, the involvement of FGFRs in the modulation of the expression of HPV-derived oncogenes [210,211].

bFGF secretion by CAF induces phosphorylation of the FGFR4 receptor subtype, which promotes tumor cell progression and also involves MAPK/ERK1/2 signaling. This suggests that the bFGF–FGFR4 interaction could become a potential target for the treatment of ovarian cancer patients. Furthermore, considering the prognostic value of bFGF, it can be used as a highly selective marker in ovarian cancer [212].

### 4.14. Summary of Current Anticancer Therapies

Summarizing, this literature review comprehensively examined the biological function of bFGF, shedding light on its crucial role in the tumorigenesis of different types of cancer; this is why it is an attractive target for successful therapies, as summarized in Table 1.

### 4.15. Clinical Trials Evaluating FGFR Targeted Therapies

As stated above, the FGFR family is known to be an established target in cancer therapy. As these receptors are involved in angiogenesis, proliferation, and other processes that guarantee cell survival in the tumor microenvironment, several trials focusing on the FGF/FGFR axis have been performed to evaluate new therapeutic strategies.

Inhibition may be total or selective for some receptors, such as AZD4547, which inhibits FGFR 1-3 [213]. In particular, in their study Aggarwal et al., showed a tolerable dosage of AZD4547, but unfortunately, minimal efficacy in patients with the alteration of FGFR 1-3 in squamous cell lung cancer was detected.

Many of these clinical trials aim to evaluate progression-free survival (FPS), tolerability, and safety. The clinical trials reported in Table 2 show the inhibition of FGFR in different types of cancer, such as in cholangiocarcinoma [214,215,216]; in these studies, selective inhibitors of FGFR 1-4 were used, such as Futibatinib and Pemigatinib, demonstrating their efficacy. Regarding the safety, the most common adverse effect of the use of such drugs is hyperphosphatemia, probably attributable to the function of FGF23 and FGFR signaling in phosphate homeostasis.

The endpoints of these clinical trials have unfortunately not been reached or have only partially been reached, as in the case of the study performed by Konecny and colleagues [217]. In this trial, two groups of women with endometrial cancer and FGFR receptor mutations or no mutations, respectively, were involved. In that study, no patient reported a complete response, only a partial response. Indeed, only one patient with an FGFR2 mutation showed a partial clinical response. The ratio of patients who achieved an overall improvement was 5% (1 of 22 patients) in the FGFR2 mutation group and 16 (5 of 31) in the FGFR2 non-mutation group. In the end, the percentage of patients who had general well-being was 64%, and the progression-free survival was 4 months. All the clinical trials reported in the table, therefore, highlight the correlation between oncological therapy and the FGF/FGFR pathway. However, considering the small number of clinical studies, future investigations will or will not establish the efficacy of FGFR inhibitors in cancer.

**Table 2:** Clinical trials evaluating new therapies against FGF/FGFR axis in different tumors.

Cancer Type	First Author	Title	Therapeutic Treatment	References
Gastric/Gastric-oesophageal	Wainberg et al. (2022)	Bemarituzumab in patients with FGFR2b-selected gastric or gastro-oesophageal junction adenocarcinoma (FIGHT): a randomised, double-blind, placebo-controlled, phase 2 study	Treatment with Bemarituzumab effectively inhibits the pathway of FGF and FGFR.	[218]
Bile ducts	Bibeau et al. (2022)	Progression-Free Survival in Patients with Cholangiocarcinoma with or Without FGF/FGFR Alterations: A FIGHT-202 Post Hoc Analysis of Prior Systemic Therapy Response	Patients with an alteration of FGFR showed a more prolonged progression-free survival during treatment with Pemigatinib and an association with second-line treatment.	[214]
Prostate	Liow et al. (2022)	Phase 2 Study of Neoadjuvant FGFR Inhibition and Androgen Deprivation Therapy Prior to Prostatectomy	The study showed the effects of FGF/FGFR-signaling inhibition and acute androgen deprivation.	[219]
Bile ducts, breast, colon, head and neck, other solid tumors	Subbiah et al. (2022)	FIGHT-101, a first-in-human study of potent and selective FGFR 1-3 inhibitor pemigatinib in pan-cancer patients with FGF/FGFR alterations and advanced malignancies	Pemigatinib has been shown to be clinically and pharmacodynamically safe; it is also safe towards tumors due to FGFR mutation.	[215]
Lung	Aggarwal et al. (2022)	SWOG S1400D (NCT02965378), a Phase II Study of the Fibroblast Growth Factor Receptor Inhibitor AZD4547 in Previously Treated Patients with Fibroblast Growth Factor Pathway-Activated Stage IV Squamous Cell Lung Cancer (Lung-MAP Substudy)	Treatment with AZD4547 showed a safe profile but unfortunately a modest improvement in patients with FGFR mutations.	[213]
Prostate	Choi et al. (2018)	Phase II Study of Dovitinib in Patients with Castration-Resistant Prostate Cancer (KCSG-GU11-05)	This study evaluated the progression-free survival and then the safety profile of dovitinib in patients who demonstrated different alterations of FGF and VEGF receptors.	[220]
Endometrial	Konecny et al. (2015)	Second-line dovitinib (TKI258) in patients with FGFR2-mutated or FGFR2-non-mutated advanced or metastatic endometrial cancer: a non-randomised, open-label, two-group, two-stage, phase 2 study	Evaluation of the safety and activity of dovitinib, FGFR, VEGFR, PDGFR-β, and c-KIT inhibitors in patients presenting and not presenting alterations of these receptors.	[217]
Renal	Kim et al. (2011)	Phase I/II and pharmacodynamic study of dovitinib (TKI258), an inhibitor of fibroblast growth factor receptors and VEGF receptors, in patients with advanced melanoma	The study reported that the tolerable dose of dovitinib effective in inhibiting the FGFR family was 400 mg/d.	[221]
Bile ducts	Goyal et al. (2023)	Futibatinib for FGFR2-Rearranged Intrahepatic Cholangiocarcinoma	Futibatinib has proven to be a valuable treatment for patients with FGFR2 abnormalities with cholangiocarcinoma.	[216]
Glioblastoma	Lee et al. (2019)	Phase II trial of ponatinib in patients with bevacizumab-refractory glioblastoma	The aim of the study was to evaluate patient 3-month progression-free survival, overall survival, and safety with bevacizumab-resistant GBM.	[222]

## 5. Conclusions

Despite the enormous progress made by scientific research in the last few decades, the mortality rate from some forms of cancer remains high and thus constitutes a clinical challenge to overcome.

In this scenario, bFGF-targeted therapies could constitute innovative treatment options that can be assessed in preclinical studies and, in particular, in clinical trials. Moreover, diagnostic assays to quantify bFGF, FGFRs, and downstream signaling molecules could constitute an important tool to better select a target patient population for the higher efficacy of cancer therapies, as well as for personalized medicine.

## Figures and Tables

**Table 1:** The table summarizes all the bFGF/FGFR axis targeting therapies described in this review.

Cancer Type	First Author	Title	Therapeutic Treatment	References
Brain	Meric-Bernstam et al. (2022)	“Futibatinib, an Irreversible FGFR1–4 Inhibitor, in Patients with Advanced Solid Tumors Harboring FGF/FGFR Aberrations: A Phase I Dose-Expansion Study.”	Fisogatinib and Futibatinib could be two effective and safe compounds capable of improving the CNS neoplastic conditions.	[74]
	Khabibov et al. (2022)	“Signaling pathways and therapeutic approaches in glioblastoma multiforme.”		[75]
Esophageal	Shi et al. (2016)	“FGF2 regulates proliferation, migration, and invasion of ECA109 cells through PI3K/Akt signalling pathway in vitro.”	LY294002, a PI3K inhibitor, as a therapeutic strategy for alleviating the tumorigenic effects of bFGF.	[77]
Gastrointestinal	Fan et al. (2015)	“A peptide derivative serves as a fibroblast growth factor 2 antagonist in human gastric cancer.”	P29 and P32 suppressed the bFGF-induced proliferation of GC cells and inhibited the activation of AKT and Erk1/2 cascades.	[85]
	Li et al. (2016)	“Peptidomimetic suppresses proliferation and invasion of gastric cancer cells by fibroblast growth factor 2 signaling cascade blockage.”		[86]
	Gao et al. (2022)	“miRNA-381-3p Functions as a Tumor Suppressor to Inhibit Gastric Cancer by Targeting Fibroblast Growth Factor Receptor-2.”	MiR-381-3p and miR-195 target bFGF, decreasing tumorigenesis of GC.	[87]
	Wang et al. (2020)	“MicroRNA-195 inhibits human gastric cancer by directly targeting basic fibroblast growth factor.”		[88]
	Guo et al. (2012)	“Toxicarioside A inhibits SGC-7901 proliferation, migration and invasion via NF-kappaB/bFGF signaling.”	Toxicarioside A reduced cell viability, cell growth, cell migration, and invasion in SGC-7901 GC cells by NF-κB/bFGF/FGFR1 signaling.	[89]
	Zhu et. al. (2017)	“Catalpol suppressed proliferation, growth and invasion of CT26 colon cancer by inhibiting inflammation and tumor angiogenesis.”	Catalpol blocked cell proliferation, growth, and invasion of CT26 lines.	[97]
	Zhou et al. (2021)	“Oridonin inhibits tumor angiogenesis and induces vessel normalization in experimental colon cancer.”	Oridonin controlled JAK2/STAT3 signaling and angiogenesis-related factors, including bFGF.	[98]
CRC	Sudha et al. (2021)	“Pomegranate (Punica granatum) Fruit Extract Suppresses Cancer Progression and Tumor Angiogenesis of Pancreatic and Colon Cancer in Chick Chorioallantoic Membrane Model.”	Morin and pomegranate, respectively, decreased bFGF levels in vivo and in colo205 cell lines.	[99]
	Sharma et al. (2020)	“Morin supplementation modulates PERK branch of UPR and mitigates 1,2-dimethylhydrazine-induced angiogenesis and oxidative stress in the colon of experimental rats.”		[100]
Liver	Wei et al. (2017)	“Plumbagin restrains hepatocellular carcinoma angiogenesis by suppressing the migration and invasion of tumor-derived vascular endothelial cells.”	Plumbagin suppresses angiogenesis pathways such as PI3K-Akt, VEGF/KDR, and Angiopoietins/Tie2, as well as angiogenic factors such as bFGF.	[110]
	Liu et al. (2020)	“Sorafenib combined with transarterial chemoembolization prolongs survival of patients with advanced hepatocellular carcinoma.”	Sorafenib-treated HCC patients evidently revealed lower levels of VEGF, bFGF, and AFP in serum.	[111]
	Tong et al. (2021)	“The effect of TACE in combination with thalidomide-mediated adjuvant therapy on the levels of VEGF and bFGF in patients with hepatocellular carcinoma.”	Combined therapies of TACE with thalidomide downregulated VEGF and bFGF levels.	[114]
Pancreatic	Li et al. (2016)	“Celecoxib suppresses fibroblast growth factor-2 expression in pancreatic ductal adenocarcinoma PANC-1 cells.”	Quercetin-3-O-glucoside and Celecoxib acted as bFGF inhibitors, avoiding the expansion of local metastasis.	[119]
	Lee et al. (2016)	“Quercetin-3-O-glucoside suppresses pancreatic cancer cell migration induced by tumor-deteriorated growth factors in vitro.”		[120]
Oral cavity	Khandelwal et al. (2018)	“Local and systemic Curcumin C3 complex inhibits 4NQO-induced oral tumorigenesis via modulating FGF-2/FGFR-2 activation.”	Curcumin inhibited 4NQO-induced tumorigenesis via modulation of the bFGF/FGFR-2 axis.	[131]
	Aytatli et al. (2022)	“AZD4547 targets the FGFR/Akt/SOX2 axis to overcome paclitaxel resistance in head and neck cancer.”	AZD4547 targeted the FGFR/Akt/SOX2 axis to overcome paclitaxel resistance in HNSCC.	[132]
Lung	Yang et al. (2017)	“Production of bFGF monoclonal antibody and its inhibition of metastasis in Lewis lung carcinoma.”	mAb-E12 inhibited CLL metastasis spreading.	[134]
Kidney	Zhang et al. (2018)	“MiR-148b-3p inhibits renal carcinoma cell growth and pro-angiogenic phenotype of endothelial cell potentially by modulating FGF2.”	miR-148b-3p stimulated renal carcinoma cell apoptosis and migration growth via the FGF2-FGFR2 pathway.	[144]
	Xu et al. (2015)	“miR-203 inhibition of renal cancer cell proliferation, migration and invasion by targeting of FGF2.”	miR-203 targeted bFGF, limiting renal cancer cell growth and metastases.	[145]
Bone	Tsubaki et al. (2011)	“Blockade of the Ras/MEK/ERK and Ras/PI3K/Akt pathways by statins reduces the expression of bFGF, HGF, and TGF-beta as angiogenic factors in mouse osteosarcoma.”	Inhibition of growth factors such as bFGF, HGF, and TGF- β with Statins.	[154]
Thyroid	Xu et al. (2017)	“miR-27b-3p is Involved in Doxorubicin Resistance of Human Anaplastic Thyroid Cancer Cells via Targeting Peroxisome Proliferator-Activated Receptor Gamma.”	miR-195 overexpression limited bFGF and, consequently, the proliferation, migration, and invasion of PTC cell lines.	[159]
	Wu et al. (2019)	“Peptide P11 suppresses the growth of human thyroid carcinoma by inhibiting the PI3K/AKT/mTOR signaling pathway.”	P11 peptide inhibited the PI3K/Akt/mTOR signaling pathway.	
Bladder	Chen et al. (2016)	“FGF2-mediated reciprocal tumor cell-endothelial cell interplay contributes to the growth of chemoresistant cells: a potential mechanism for superficial bladder cancer recurrence.”	Evaluation of the antagonism against bFGF using the neutralizing antibody, which, however, did not affect the growth of 253J/DOX cells.	[167]
Prostate	Hotchkiss et al. (2002)	“Inhibition of endothelial cell function in vitro and angiogenesis in vivo by docetaxel (Taxotere): association with impaired repositioning of the microtubule organizing center.”	Taxane and Docetaxel effectively downregulated bFGF expression.	[191]
	Cenni et al. (2007)	“Inhibition of angiogenesis via FGF-2 blockage in primitive and bone metastatic renal cell carcinoma.”	Antisense bFGF oligonucleotide blocked the production of FGF by both tumor and endothelial cells.	[194]
Breast	Shee et al. (2018)	“Therapeutically targeting tumor microenvironment-mediated drug resistance in estrogen receptor-positive breast cancer.”	PD170374 inhibiting bFGF stopped ECM/FGF-mediated regulation of ER breast cancer.	[203]
Ovarian	Wang et al. (2021)	“Cancer-associated fibroblasts secrete FGF-1 to promote ovarian proliferation, migration, and invasion through the activation of FGF-1/FGFR4 signaling.”	PD173074 terminated cellular proliferation, migration, and invasion.	[209]

## Data Availability

Not applicable.
